# Measuring the Caregiver Burden of Caring for Community-Residing People with Alzheimer’s Disease

**DOI:** 10.1371/journal.pone.0132168

**Published:** 2015-07-08

**Authors:** Hongmei Yu, Xiaocheng Wang, Runlian He, Ruifeng Liang, Liye Zhou

**Affiliations:** 1 Department of Health Statistics, School of Public Health, Shanxi Medical University, Taiyuan, China; 2 Department of Medical Record Management, Shanxi Provincial People’s Hospital, Taiyuan, China; 3 Department of Nursing, Taiyuan Central Hospital, Taiyuan, Shanxi, P. R. China; 4 Department of Environmental Health, School of Public Health, Shanxi Medical University, Taiyuan, China; 5 Department of Mathematics, School of Basic Medical Sciences, Shanxi Medical University, Taiyuan, China; Nathan Kline Institute and New York University School of Medicine, UNITED STATES

## Abstract

**Objectives:**

To assess the direct and indirect effects of patient or caregiver factors on caregiver burden of caring for community-residing people with mild Alzheimer’s disease (AD).

**Methods:**

We conducted a cross-sectional study of patients diagnosed with AD from two hospitals and three communities in Taiyuan, China and their caregivers. For this survey, 200 patients with mild AD and their caregivers were selected. Caregivers were asked to provide sociodemographic information including age, gender, relationship with the patient, level of education, and number of contact hours per week with the patient. Caregiver burden was assessed using the Caregivers Burden Inventory. The caregivers also completed other measures including the Positive Aspects of Caregiving, the Family Adaptation, Partnership, Growth, Affection, and Resolve, and the Social Support Rating Scale. The patients with AD completed the Montreal Cognitive Assessment; their caregivers completed the Activities of Daily Living Scale and a questionnaire about the patients’ Behavioral and Psychological symptoms of Dementia. The main outcome in this study was caregiver burden. The care receivers’ level of cognitive function, physical function, and behavioral problems were treated as original stress; the primary appraisal variable was measured as the number of hours of caregiving in the previous week reported by the caregiver. Mediator variables included perceived social support, family function, and caregiving experience. Path analysis was used to build the interrelationship among caregiver burden and patient or caregiver factors.

**Results:**

A lower level of cognitive function in patients (*r* = −0.28, *p*<0.001) and longer hours of caregiving (*r* = 0.17, *p* = 0.019) were related to increased caregiver burden. Greater social support (*r* = −0.23, *p*<0.001), family function (*r* = −0.17, *p* = 0.015) and caregiving experience (*r* = −0.16, *p* = 0.012) were related to decreased caregiver burden. Social support (*r* = 0.16, *p* = 0.040) and family function (*r* = 0.25, *p* = 0.002) were directly related to patients’ level of cognitive functioning, but were mediator factors between level of cognitive function in patients and caregiver burden. Similarly, social support was a mediator factor between patients' daily function (*r* = −0.23, *p* = 0.004) and caregiver burden; while caregiving experience mediated the link between behavioral and psychological symptoms in patients (*r* = 0.36, *p*<0.001) and caregiver burden.

**Conclusion:**

Level of cognitive function and hours of caregiving were directly related to caregiver's burden. Social support, family function and caregiving experience could mediate the relationship between patient factors and caregiver burden. Focusing on patient factors and promoting caregiver care will be helpful in lowering the perceived burden of caregiving.

## Introduction

Dementia is expected to become a serious health and social burden of disease in the older adult population given its naturally progressive and irreversible course. The problem is especially severe in low-to-middle-income countries (LMICs), where dementia is the most important independent contributor to disability in the elderly and resources to diagnose and treat dementia are limited. By the mid-21st century, 78% of the world’s older adult population will reside in LMICs, with expected concomitant increases in dementia cases [1]. The most common type of dementia, accounting for 60–80% of all cases, is that resulting from Alzheimer’s disease (AD) [2].

LMICs are characterized by low levels of awareness regarding dementia as a chronic degenerative brain syndrome, and by an absence of supportive health and welfare services. Almost all patients with dementia are cared for at home by a co-resident family member. This situation is unlikely to change in the near future, as institutional care is neither affordable nor culturally acceptable. It is important to realize that AD not only affects the patient, but also the whole family and particularly the caregiver [3]. Providing care for people with AD is particularly demanding as the needs for care escalate with the progression of the disease. Because caregivers play such vital roles for people with dementia, it is critical to understand the factors that affect their caregiver burden. George and Gwyther [4] defined caregiver burden as “the physical, psychological or emotional, social, and financial problems that can be experienced by family members or friends who care for impaired older adults.” Stull et al [5] concluded that caregiver burden is a unique domain of the caregiving experience.

**Fig 1:**
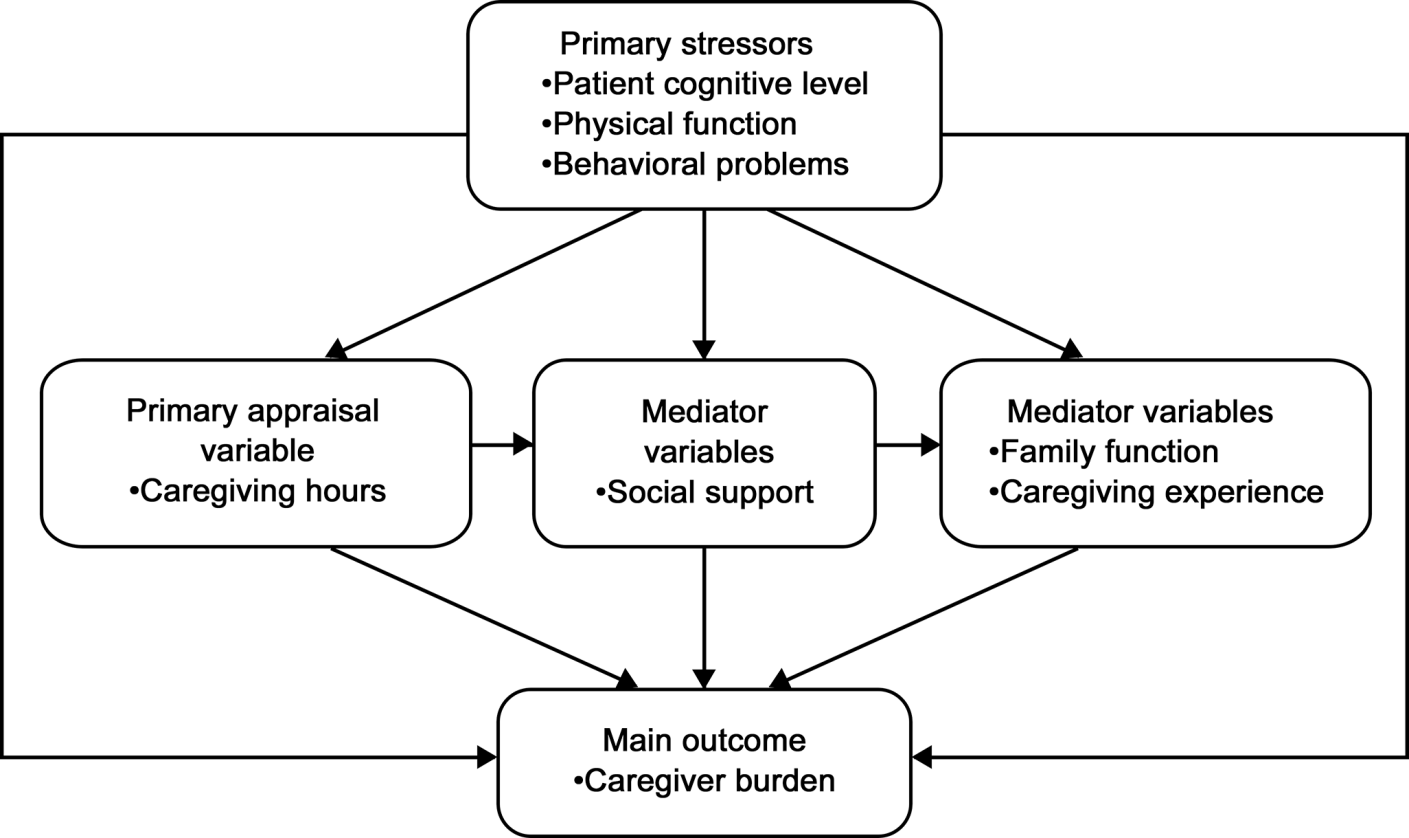
Conceptual model. Based on Yates et al. (1999) and Chappell and Reid (2002).

Identifying possible predictive factors of perceived burden among caregivers of AD patients could improve integrated healthcare strategies for this type of illness. A number of variables, including the caregiver’s sociodemographic characteristics, the clinical characteristics of the patient’s illness, and the social support and personal resources available to the caregiver determine the perceived burden of caregiving [6–8]. A version of the caregiver stress/appraisal model was proposed by Yates et al.[9] and assessed by Chappell and Reid [10]. Currently there is no consensus regarding the predictors of high levels of burden of AD caregivers especially those caring for patients with mild dementia. We used an adaptive version of the stress-appraisal model of Chappell and Reid and assumed that: (1) primary caregiver-stressors (cognitive impairments, functional disability, and problem behaviors) lead directly and indirectly to caregiver burden; (2) this indirect relationship is mediated by one of the three mediator variables: perceived social support, family function, and caregiving experience; (3) the number of caregiving hours is treated as the primary appraisal variable. Fig 1 represents this conceptual model.

Based on the adaptive stress-appraisal model of Chappell and Reid, the primary aim of this study was to further explore the caregiver burden of caregivers of community-residing people with mild AD and the relationships between caregiver-stressors (cognitive impairment, functional dependency of the recipient, and behavioral symptoms), appraisal (informal hours of caring), caregiver factors (social support, family function, and caregiving experience) and their effects on the burden of AD caregivers in mainland China.

## Methods

### Study participants

We conducted a cross-sectional study of patients diagnosed with AD from two hospitals and three communities in Taiyuan, China and their caregivers. Patients were included if they were outpatients diagnosed with mild AD based on criteria established in the Diagnostic and Statistical Manual of Mental Disorders, 5th edition [11] and were aged at least 60 years. Patients were excluded from the study if they had malignant or severe organic or psychiatric diseases that made it impossible to complete the study questionnaires, or if they did not provide inform consent.

Each patient was asked to identify his or her primary caregivers. We recruited those caregivers who met the definition of a family caregiver established by Perlick et al. [12]. They defined a primary caregiver as a person who fulfills at least three of the following criteria: (1) a parent, partner, or other relative; (2) maintains frequent contact with the patient; (3) provides significant financial support to the patient; (4) is the person who is most often present with the patient during consultation or treatment and who is aware of the severity of the illness (accompanies the patient to medical appointments, participates in consultations and therapy, supervises eating behavior at home, etc.); (5) is the person the therapy team contacts in the event of an emergency. The exclusion criteria for the caregivers were the same as for the patients.

The study was approved by the ethics committee of Shanxi Medical University and participants provided their written informed consent to participate in this study. Data collection started in August 2013. Patients and caregivers were informed of the study objectives and data confidentiality. When both the patient and the caregiver gave their informed consent to participate, a structured face to face interview was conducted separately by professional survey interviewers to collect data using the study instruments. Average survey completion time was 1 hours 20 minutes, ranging from 50 minutes to 2 hours. Quality control procedures involved checking the accuracy of responses recorded by interviewers; there was no evidence of any problems.

### Measures

Caregivers were asked to provide sociodemographic information including age, gender, relationship with the patient (partners, children, etc.), level of education, whether he or she lived with the patient, number of contact hours per week with the patient, and health status. Caregivers were also asked to complete four instruments to assess their burden of caregiving, perception of caregiving, family function and social support. We used versions of these instruments that have been validated in Chinese.

The Caregiver Burden Inventory (CBI) is a 24-item multi-dimensional questionnaire measuring caregiver burden with five subscales, namely, time dependence, developmental, physical, social, and emotional burden [13]. Scores for each item are evaluated using a five-point Likert scale ranging from 0 (not at all disruptive) to 4 (very disruptive) and all of the scores on the 24-item scale are summed with higher scores indicating higher burden.

The Positive Aspects of Caregiving (PAC), a nine-item scale, presents statements about a caregiver’s mental or affective state in the context of the caregiving experience [14]. Responses were provided on a five-point Likert scale (agree/disagree) and were designed to assess the perception of benefits within the caregiving context, such as feeling useful, feeling appreciated, and finding meaning. Scores ranged from 9 to 45, with higher scores indicating more positive caregiving appraisals.

The Family Adaptation, Partnership, Growth, Affection, and Resolve (APGAR) Index is designed to test the five areas of family function listed above [15]. Adaptation is the utilization of intra- and extra-familial resources for problem solving when family equilibrium is disrupted during a crisis. Partnership is the sharing of decision making and nurturing responsibilities by family members. Growth is the physical and emotional maturation and self-fulfillment that is achieved by family members through mutual support and guidance. Affection is the caring or loving relationship that exists among family members. Resolve is the commitment to devote time to other members of the family for physical and emotional nurturing. It also usually involves a decision to share wealth and space. Scores for each item range from 0 to 2, and the scores for each of the five questions are then totaled with higher scores indicating a higher level of family function.

The Social Support Rating Scale (SSRS) was developed by Xiao between 1986 and 1993 for assessing social support [16]. It is a 10-item measure: three items for evaluating objective support, four for subjective support, and three for social support availability. Higher scores indicate a higher level of social support.

AD patients were asked to provide basic demographic data, including age, gender, marital status, and educational level. There were three instruments that were used to assess their activities of daily living, cognitive status and behavioral pathology.

The Activities of Daily Living (ADL) Scale measures two important domains of functioning in older people: the Physical Self-Maintenance Scale (PSMS), which assesses self-care ability in areas of toileting, feeding, dressing, grooming, locomotion, and bathing; and the Instrumental Activities of Daily Living (IADL) Scale, which assesses a somewhat more complex set of behaviors including telephoning, shopping, food preparation, housekeeping, laundering, use of transportation, use of medicine, and financial behavior [17–18]. Caregivers completed the ADL Chinese version; scores for each item range from 1 (independent) to 4 (does not do) with higher scores indicating more functional problems.

The Montreal Cognitive Assessment (MoCA), which is one of the most widely used brief screening instruments for cognitive impairment, provides a total score ranging from 0 to 30, with lower scores indicating greater cognitive impairment [19]. The MoCA does not focus on a single cognitive domain, but it measures each domain that may be influenced by cognitive status. Thus, in this investigation, the MoCA Chinese version was used to assess level of cognitive impairment of AD patients.

The frequency and severity of behavioral and psychological symptoms of dementia (BPSD) were assessed using the Behavioral Pathology in Alzheimer's Disease Rating Scale (BEHAVE-AD), which measures behavioral and psychological symptoms in seven symptom domains (paranoid and delusional ideation, hallucinations, activity disturbances, aggressiveness, diurnal rhythm disturbances, affective disturbance, and anxieties and phobias) [20]. BEHAVE-AD is scored on a four-point scale according to disease severity. We used the total score for all symptom domains with higher scores indicating more serious behavioral and psychological symptoms. BEHAVE-AD was administered to caregivers for completion.

The means, standard deviations (SD), minimum, maximum, Cronbach’s alpha, and possible scale score ranges for all of the measures in the analyses can be found in Table 1.

**Table 1:** Variable Means, Standard Deviations, Minimum, Maximum, Cronbach’s Alpha, and Possible Scale Score Ranges.

Variable	Measure	Mean	Standard Deviation	Minimum	Maximum	Cronbach’s Alpha	Possible scale score ranges
Cognitive impairment	MoCA	16.11	5.29	3	25	0.805	Lower: more cognitive impairment (0–30)
Functional problems	ADL	27.02	10.27	12	51	0.648	Higher: more functional problems (14–56)
Behavior problems	BPSD	15.14	8.59	2	37	0.703	Higher: more behavior problems (0–75)
Social support	SSRS	35.93	8.75	20	61	0.745	Higher: more perceived support (11–64)
Family function	APGAR	6.05	2.16	1	10	0.756	Higher: grater family function (0–10)
Caregiving experience	PAC	29.73	8.65	10	45	0.579	Higher: more perceived positive state (9–45)
Caregiver burden	CBI	47.54	17.61	17	92	0.812	Higher: greater caregiver burden (0–96)

MoCA = Montreal Cognitive Assessment; ADL = Activities of Daily Living; BPSD = Behavioral and Psychological Symptoms of Dementia; SSRS = Social Support Rating Scale; APGAR = Adaptation, Partnership, Growth, Affection, and Resolve; PAC = Positive Aspects of Caregiving; CBI = Caregiver Burden Inventory.

### Statistical analysis

Descriptive statistics of sociodemographic variables and scales were calculated using means and SD for quantitative data, and frequencies and percentages for categorical variables. Cronbach’s alpha coefficient was used to assess the internal consistency of the scales used and alpha coefficients equal to or greater than 0.70 were considered to be satisfactory.

The main outcome in this study was caregiver burden. Care receiver level of cognitive function, physical function, and behavioral problems were treated as original primary stressors; the primary appraisal variable was measured as the number of caregiving hours in the previous week reported by the caregiver. Mediator variables included perceived social support, family function, and caregiving experience (Fig 1). Path analysis was used to build the interrelationships among caregiver burden and patient or caregiver factors. The model used was based on a previously proposed stress/appraisal path model and the conceptual model was tested using ordinary least squares regression coefficient estimates (standardized) in a path analysis. All data analyses were computed using SPSS 13.0 and AMOS 17.0.

## Results

### Patient and caregiver characteristics

During the study period, 200 patients with mild AD and their respective caregivers were recruited. Of these, 168 (84%) caregiver/care recipient dyads fulfilled the criteria and completed all the questionnaires. The sociodemographic characteristics of the caregivers and patients with AD are shown in Table 2. Among the 168 caregivers of AD patients, 68.5% were females and the mean age of caregivers was 56.8 years (SD 13.8). The relationship between caregiver and patient was as follows: 42.3% were partners, 45.8% siblings or children, and 11.9% were identified as “other”. Of the 168 AD patients, 52.4% were females and the mean age of patients was 73.3 years (SD 7.6). The mean number of informal hours of care provided weekly by each caregiver was 62.8 hr (SD 32.5), with a range of 10 to 142. When comparing patients and caregivers who agreed to participate and those who declined to participate, all variables were equally distributed between the groups.

**Table 2:** Sociodemographic Characteristics of Patients and Caregivers.

Variable	N (%)	Variable	N (%)
Patients		Caregivers	
Gender		Gender	
Male	80 (47.6)	Male	53 (31.5)
Female	88 (52.4)	Female	115 (68.5)
Age (years)		Age (years)	
60~	55 (32.7)	20~	17 (10.1)
70~	79 (47.0)	40~	77 (45.8)
≥80	34 (20.2)	≥60	74 (44.0)
Marital status		Relationship with patients	
Married	132 (78.6)	Partners	71 (42.3)
Widowed	36 (21.4)	Children	77 (45.8)
Educational level		Other	20 (11.9)
≤6 years	55 (32.7)	Health status	
7–9 years	81 (48.2)	Good	109 (64.9)
≥10 years	32 (19.0)	Poor	59 (35.1)

### Caregiver burden and its relationship to patient or caregiver factors

**Fig 2:**
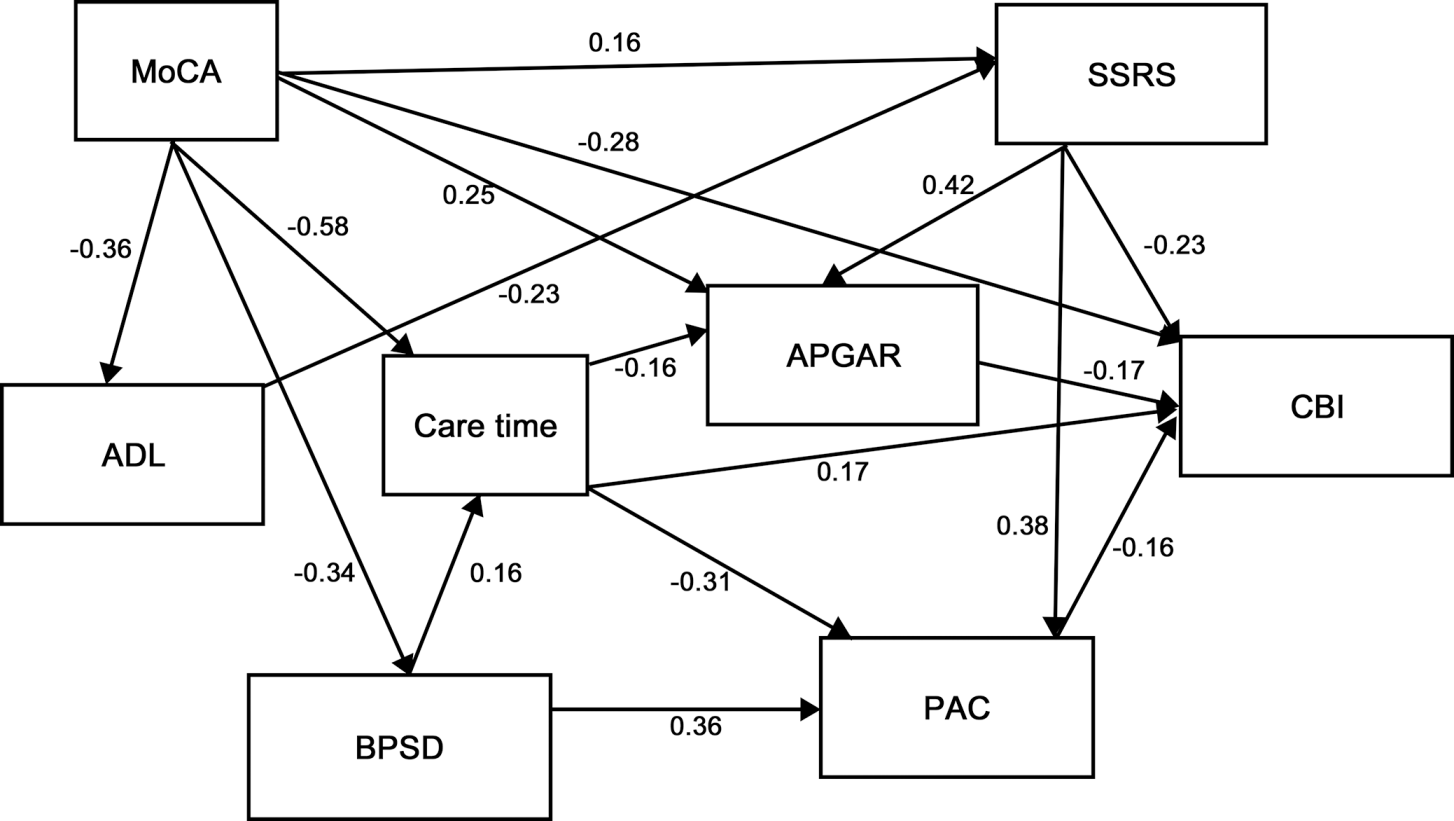
Path analysis plot with standardized coefficients. Only significant coefficients are shown. MoCA = Montreal Cognitive Assessment; ADL = Activities of Daily Living; BPSD = Behavioral and Psychological Symptoms of Dementia; SSRS = Social Support Rating Scale; APGAR = Adaptation, Partnership, Growth, Affection, and Resolve; PAC = Positive Aspects of Caregiving; CBI = Caregiver Burden Inventory.

The results showed that the mean CBI score was 47.5 (SD 17.6) at a medium level. The final path model is shown in Table 3 and Fig 2. For the primary stressors, cognitive function had statistically significant direct effects on CBI scores when controlling for contextual variables and simultaneously controlling for all other variables in the model (including care recipient’s age in years, gender, marital status, educational level, caregiver’s age, gender, and relationship with patients). Cognitive function in patients was negatively associated with CBI scores (*r* = −0.28, *p*<0.001), with a lower level of cognitive function in patients leading to higher CBI scores. The other two primary stressors including activities of daily living and behavioral problems were not directly associated with caregiver burden. Among the primary stressors, functional dependency and behavioral problems were predicted by cognitive status, with cognitively impaired care recipients reporting higher levels of ADL dependency (*r* = -0.36, *p*<0.001) and more behavioral problems (*r* = −0.34, *p*<0.001).

**Table 3:** Regression Estimates (with beta values).

Path	Regression coefficient	Standard error	*P*	Standard regression coefficient
ADL ← MoCA	−0.69	0.140	<0.001	−0.36
BPSD ← MoCA	−0.57	0.121	<0.001	−0.34
SSRS ← MoCA	0.26	0.129	0.040	0.16
SSRS ← ADL	−0.19	0.067	0.004	−0.23
Hours caregiving ← MoCA	−3.57	0.379	<0.001	−0.58
Hours caregiving ← BPSD	0.56	0.228	0.009	0.16
PAC ← BPSD	0.35	0.068	<0.001	0.36
PAC ← Hours caregiving	−0.08	0.019	<0.001	−0.31
PAC ← SSRS	0.37	0.065	<0.001	0.38
APGAR ← MoCA	0.10	0.032	0.002	0.25
APGAR ← SSRS	0.10	0.015	<0.001	0.42
APGAR ← Hours caregiving	−0.01	0.005	0.041	−0.16
CBI ← APGAR	−1.38	0.571	0.015	−0.17
CBI ← PAC	−0.32	0.125	0.012	−0.16
CBI ← MoCA	−0.91	0.246	<0.001	−0.28
CBI ← SSRS	−0.47	0.134	<0.001	−0.23
CBI ← Hours caregiving	0.09	0.039	0.019	0.17

MoCA = Montreal Cognitive Assessment; ADL = Activities of Daily Living; BPSD = Behavioral and Psychological Symptoms of Dementia; SSRS = Social Support Rating Scale; APGAR = Adaptation, Partnership, Growth, Affection, and Resolve; PAC = Positive Aspects of Caregiving; CBI = Caregiver Burden Inventory.

Regarding the primary appraisal variable, hours of caregiving was positively and directly related to CBI scores (*r* = 0.17, *p* = 0.019), with longer hours of caregiving leading directly to higher reported CBI scores. The primary appraisal variable was predicted by two of the primary stressors, cognitive status (*r* = −0.58, *p*<0.001) and behavioral problems (*r* = 0.16, *p* = 0.009). Also, hours of caregiving was negatively related to perception of caregiving (*r* = −0.31, *p*<0.001) and family function (*r* = −0.16, *p* = 0.041).

Among the mediator variables, increased social support (*r* = −0.23, *p*<0.001), family function (*r* = −0.17, *p* = 0.015) and caregiving experience (*r* = −0.16, *p* = 0.012) were related to decreased caregiver burden. Social support (*r* = 0.16, *p* = 0.040) and family function (*r* = 0.25, *p* = 0.002) were directly and positively influenced by patients’ level of cognitive functioning, but were mediator factors between level of cognitive function in patients and caregiver burden. Similarly, social support was the mediator factor between patients' daily function (*r* = -0.23, *p* = 0.004) and caregiver burden; while caregiving experience mediated behavioral and psychological symptoms in patients (*r* = 0.36, *p*<0.001) and caregiver burden.

## Discussion

It is estimated that over 100 million people will be affected by AD by 2050. Family members commonly fulfill the role of caregiver out of love, respect, commitment, and/or a sense of duty or responsibility for the care recipient. The level of caregiver burden that is experienced by caregivers can be affected by the culture to which they belong. In Chinese culture, the caregiver commonly does not differentiate their caregiving role from their other daily activities; they view caregiving as just another part of family life. Furthermore, Chinese caregivers of patients with dementia had higher scores on measures of depression and caregiver burden compared with caregivers of dementia patients in Western societies [21].This study attempted to capture the complexities involved in caregiving through the use of a path model and to measure the effects of the variables in that model on caregiver burden. Identifying modifiable, easily measured factors in a caregiver’s or patient’s profile could help alleviate burden and improve both the care given to the patient with AD and the caregiving experience.

### Caregiver burden and caregiver-stressors

For the three primary caregiver-stressors (cognitive impairments, functional disability, and problem behaviors), the relationship between cognitive impairment and caregiver burden is less clear; studies indicate either a positive correlation [22,23] or no direct relation [24,25]. Our study showed that care recipient’s cognitive status was directly associated with caregiver burden and directly related to functional dependency, behavioral problems, hours of informal care, and finally indirectly associated with caregiver burden. Thus, it is the cognitive status of the care recipients’ that determines the associated burden, suggesting using care recipient cognitive status as a starting point when considering caregiver burden and stressors. Our study implies that an intervention aimed at slowing the progression of the dementia may have a positive effect on caregiver burden.

The lack of an observed direct relationship between the other two primary stressors and caregiver burden in this study is unusual and, in fact, counter to most research in this area. Loss of abilities associated with AD causes a variety of changes in personality, emotion, behavior, and function [26]. These progressive and unpredictable disorders are often difficult for caregivers to manage and can cause stress, frustration, and burden [26]. Behavioral and psychological symptoms of dementia were significantly associated with increased caregiver burden [27,28]. Furthermore, behavioral disturbances of patients with dementia are one of the largest factors contributing to caregiver burden [29,30]. One study, however, showed that severity of behavioral problems was not associated with higher levels of caregiver burden [31]. The fact that we had only mild dementia patients may explain the non-significant direct relationship between physical function, behavioral problems, and caregiver burden in this study. Thus further research on patients’ physical function or behavioral problems and their relation to caregiver burden is needed.

### Caregiver burden and primary appraisal variable

Regarding the primary appraisal variable, number of hours of informal care was found to have a direct positive effect on caregiver burden in our study. Not surprisingly, an increase in informal hours of care leads directly to a greater burden [32,33]. Arai found that those caregivers who were temporarily relieved of caregiving for 3 or more hours a day were less likely to experience ‘heavier’ caregiver burden than those who were not [32]. Chou et al found that the demands of care on the caregiver had direct positive effects on caregiving involvement, and that caregiving involvement then had direct positive effects on caregiving burden [33]. These results demonstrate that use of adult day care by caregivers of patients with dementia and establishing a network of community services results in fewer caregiving hours and lower levels of caregiving-related burden [34].

### Caregiver burden and mediator variables

Social support was directly negatively related to caregiver burden, and was found to mediate level of cognitive function in patients or patients' daily function and caregiver burden. Our finding that social support was negatively related to caregiver burden is not consistent with those of Yates and associates [9], who reported that perceived emotional support from family and friends was directly related to depression but not to overload (burden). Aguglia and Shurgot found that perceived positive social support was inversely related to caregiver burden [35,36]. The amount of caregiver burden was found to be less when the patient with dementia received more visits by other relatives [31]. Li and Sprague found that caregivers required help and assistance from family members as well as their expressed encouragement and appreciation for the caregivers’ work in order to lessen the degree of burden [37].

We found that family function mediated level of cognitive function in patients and caregiver burden; this suggests relationships between poor cognitive function of the care recipient, poor family functioning and increased caregiver burden. Caregivers who reported poor family functioning also reported higher caregiver burden [38,39]. Even after controlling for caregiver depression, caregiver anxiety, and frequency of memory/behavior problems in dementia patients, poorer family functioning continued to be associated with increased levels of caregiver burden [40].

Our results suggest the importance of providing support to caregivers as a critical step in the community care of older adults with dementia. They also highlight the importance of including a family assessment and intervention when working with dementia caregivers.

We also found that caregiving experience mediated the link between behavioral or psychological symptoms in patients and caregiver burden; also, positive perception of caregiving was negatively related to caregiver burden. Recognition of positive experiences in caregiving means that providing care increases caregivers’ feelings of pride in their ability to meet challenges, improves their sense of self-worth, leads to greater closeness in relationships, and provides an enhanced sense of meaning, warmth, and pleasure [41,42]. Positive feelings are related to high levels of resilience to stress, subjective health status, and health-related quality of life [43]. Ji et al. showed that strong feelings for the patients and positive emotions arising from providing care were the main motivators of female family caregivers of patients with dementia [44].

There are several limitations of this study that are important to consider. Given the cross-sectional nature of our study, we cannot make any statements about causation; our data only reflect an impact on caregiver burden for caregivers of patients with AD. It would be useful to conduct a longitudinal study to assess whether there are changes in caregivers’ burden over time and to identify variables that may influence those changes. Another limitation is that we did not examine additional factors that may account for variations in caregivers’ experience of burden, such as level of satisfaction with other aspects of life, caregiving self-efficacy or coping strategies used and the quality of the relationship between the caregiver and the care-recipient [45]. Also, cultural factors, economic situations, and other sources of caregiver stress were not controlled in this study. The patients with dementia in this sample had mild dementia and we did not study caregiver burden related to the clinical stage of dementia. Furthermore, the sample was nonrandomized and the possibility of selection bias cannot be totally excluded, limiting the generalization of the study. The caregiving process is clearly an inherently complex one and is in need of more research.

Our study has several strengths and advantages compared to previous studies: (1) our use of validated instruments that enable the easy collection of data in an epidemiological study; (2) both caregiver and patient factors were evaluated; (3) the empirically tested model on which our study was based and adapted; (4) the path analysis model that we used to clarify direct and indirect effects of caregiver and patient factors on caregiver burden.

In summary, the findings of this study confirm the presence of a medium degree of caregiver burden in caregivers of patients with mild AD. We found that level of cognitive functioning of the care recipient and hours of caregiving were directly related to caregiver burden. Social support, family function and caregiving experience could mediate the relationship between patient factors and caregiver burden. To be effective, dementia care services in developing countries need to focus on management of AD at home. Development of a low cost, effective and sustainable dementia care service should be given due consideration by policy makers in the developing world. It is necessary to slow the progression of dementia, to provide effective support to caregivers, to enhance caregivers’ feelings of positive emotions, and to establish specialized care institutions for patients with dementia, so as to reduce caregiver burden.
